# Pharmacologic Treatment with GABA_B_ Receptor Agonist of Methamphetamine-Induced Cognitive Impairment in Mice

**DOI:** 10.2174/157015911795016976

**Published:** 2011-03

**Authors:** Hiroyuki Mizoguchi, Kiyofumi Yamada

**Affiliations:** 1 Futuristic Environmental Simulation Center, Research Institute of Environmental Medicine, Nagoya University, Nagoya 464-8601, Japan; 2 Department of Neuropsychopharmacology and Hospital Pharmacy, Nagoya University Graduate School of Medicine, Nagoya 466-8560, Japan; 3 JST, CREST, Japan

**Keywords:** Baclofen, methamphetamine, cognition, prepulse inhibition, GABA_B_ receptor.

## Abstract

Methamphetamine (METH) is a highly addictive drug, and addiction to METH has increased to epidemic proportions worldwide. Chronic use of METH causes psychiatric symptoms, such as hallucinations and delusions, and long-term cognitive deficits, which are indistinguishable from paranoid schizophrenia. The GABA receptor system is known to play a significant role in modulating the dopaminergic neuronal system, which is related to behavioral changes induced by drug abuse. However, few studies have investigated the effects of GABA receptor agonists on cognitive deficits induced by METH. In the present review, we show that baclofen, a GABA receptor agonist, is effective in treating METH-induced impairment of object recognition memory and prepulse inhibition (PPI) of the startle reflex, a measure of sensorimotor gating in mice. Acute and repeated treatment with METH induced a significant impairment of PPI. Furthermore, repeated but not acute treatment of METH resulted in a long-lasting deficit of object recognition memory. Baclofen, a GABA_B_ receptor agonist, dose-dependently ameliorated the METH-induced PPI deficits and object recognition memory impairment in mice. On the other hand, THIP, a GABA_A_ receptor agonist, had no effect on METH-induced cognitive deficits. These results suggest that GABA_B_ receptors may constitute a putative new target in treating cognitive deficits in chronic METH users.

## METHAMPHETAMINE-INDUCED COGNITIVE IMPAIRMENT

Methamphetamine (METH) increases the amount of dopamine released in synapses by reversing the function of the dopamine transporter, which is associated with the rewarding effects of the drug [1-4]. Chronic use of METH leads to addiction (dependence) and long-lasting impairment of brain function with hallucinations and delusions, which are indistinguishable from paranoid schizophrenia [5, 6]. Furthermore, chronic METH users show significantly poorer performances on measures of attention/psychomotor speed, verbal learning and memory, and executive function, demonstrating that METH dependence is associated with impairments across a range of neurocognitive domains [7].

Numerous studies indicate that METH disrupts neurotransmitter function and in particular the dopaminergic system, although changes in serotonergic, noradrenergic, and glutamatergic functions are also observed [5, 8, 9]. It has been argued that these neuropathological changes underpin the neurocognitive deficits associated with METH use in humans [5]. Consistent with this possibility, in a recent meta-analysis of the neurocognitive effects of METH, Scott *et al.* [10] reported significant impairment in several cognitive domains that are considered to affect the integrity of these neural substrates, including retrospective memory, information processing speed, and executive operations such as inhibitory control.

In order to understand the etiology of METH-induced cognitive impairment in chronic METH users, it is necessary to establish animal models. Therefore, we investigated the effect of METH on learning and memory as well as sensorimotor gating in rodents. We demonstrated that repeated METH treatment of mice followed by withdrawal impairs long-term recognition memory, without affecting learning or short-term memory, and that METH-induced cognitive impairment is reversed by an atypical antipsychotic, clozapine, but not by haloperidol [11]. Moreover, we have demonstrated that repeated METH treatment in rats impairs working memory in a delayed spatial win-shift task using a radial arm maze and that clozapine, but not haloperidol, is effective in improving the METH-induced working memory deficit [12]. Because clinical evidence has shown that clozapine is superior to typical therapeutics such as haloperidol in improving cognitive deficits in schizophrenic patients [13, 14], the METH-induced cognitive impairment in rodents may be useful as an animal model for cognitive deficits in METH abusers as well as schizophrenic patients, in which cognitive deficits are regarded as a core feature.

Our recent studies indicated that activation of the ERK1/2 signaling pathway, which is associated with the dopamine D1 receptor [15, 16], plays an important role in memory function under physiological and pathological conditions [11, 17-19]. We have also proposed that impairment of the dopamine D1 receptor-ERK1/2 signaling pathway in the prefrontal cortex (PFC) is involved in METH-induced deficits of recognition memory [11, 20], and that ERK1/2 activation is related to the performance in working memory [19]. On the basis of our findings, Ito *et al.* [17] and Mizoguchi *et al.* [21] showed that ZSET1446, a novel azaindolizine derivative, or minocycline ameliorated METH-induced cognitive impairment through, at least in part, activation of the ERK1/2 pathway in the PFC linked to dopamine D1 receptors.

## GABA RECEPTOR

The GABA receptor system is known to play a significant role in modulating the dopamine system [22]. Baclofen, a GABA_B_ receptor agonist, is known to stabilize the firing pattern of dopamine neurons [23]. Baclofen has been shown to attenuate amphetamine-induced increase in dopamine levels in the nucleus accumbens [24], and GABA_A_ receptors on dopamine neurons in the ventral tegmental area play a significant role in attenuating the effects of drug abuse in a similar manner to that of GABA_B_ receptors [25]. Therefore, several studies have demonstrated that GABA receptor agonists can inhibit the effects of drug abuse. For example, previous studies showed that baclofen reduced the reinforcing effects of many substances of abuse, such as cocaine, nicotine, heroin, and alcohol [26], possibly through GABA_B_-mediated modulation of mesolimbic dopamine transmission [27]. It was demonstrated that chronic coadministration of baclofen and amphetamine blocked the development of sensitization to the locomotor stimulation effect of amphetamine [28], and acute treatment with baclofen inhibited the expression of amphetamine-induced locomotor sensitization [29]. Moreover, a recent study showed that acute treatment with baclofen ameliorated ethanol-induced memory deficit in mice [30].

Clinical studies have shown significant haplotype associations between different genes and METH use or the development of METH-induced psychosis. In particular, several case-control association studies suggest that the human GABA_A_ receptor γ2 subunit gene is marginally associated with METH use disorder and may be one of the susceptibility genes of METH use disorder [31, 32]. Moreover, several clinical studies have demonstrated that GABA agonists including topiramate, baclofen and GABA transaminase inhibitor show promise in reducing the METH use/craving [33]. Thus, in many studies, the effects of GABA receptor agonists on hyperdopaminergic conditions induced by psychostimulant drugs have been examined; however, few studies have involved investigation of the effects of GABA receptor agonists on cognitive deficits induced by drugs abuse. In the following sections, we discuss the effects of GABA receptor agonists on METH-induced cognitive impairment.

## EFFECTS OF GABA RECEPTOR AGONISTS ON METH-INDUCED IMPAIRMENT OF RECOGNITION MEMORY

As described above, repeated treatment with METH (1 mg/kg, s.c.) for 7 days impairs object recognition memory in mice in a novel objective recognition test, which is associated with dysfunction of the dopamine D_1_ receptor-ERK1/2 pathway in the prefrontal cortex [11]. To develop novel pharmacotherapy for cognitive deficits in METH abusers, we examined the effects of GABA_A_ and GABA_B_ receptor agonists in this animal model.

We found that acute treatment with baclofen (1-2 mg/kg) improved METH-induced cognitive deficit without affecting motor function. In contrast, gaboxadol (1-3 mg/kg), a GABA_A_ receptor agonist, had no effect on METH-induced cognitive deficits. These results suggest that GABA_B_ receptor agonists may be useful for the treatment of cognitive deficit in METH abusers (Table **1**) [34]. Although further studies are necessary to clarify the molecular mechanisms of the action of baclofen, its ameliorating effect on METH-induced cognitive deficit may be, at least in part, due to the inhibitory effect on METH-evoked hyperphosphorylation of ERK1/2 in the PFC. Alternatively, a previous study demonstrated that activation of GABA_B_ receptors led to an increase in ERK1/2 phosphorylation in the CA1 area of mouse hippocampal slices and promoted CREB2-mediated transcription through an ERK-dependent mechanism, suggesting that GABA_B_ receptors may play a crucial role in regulating synaptic facilitation and memory through regulating protein synthesis and gene expression [35].

## EFFECTS OF GABA RECEPTOR AGONISTS ON METH-INDUCED DISRUPTION OF PREPULSE INHIBITION

Prepulse inhibition (PPI) of the startle reflex is viewed as a measure of a process called ‘sensorimotor gating’. Deficits in PPI are observed in patients suffering from certain psychiatric disorders such as schizophrenia [36, 37]. We have previously demonstrated that GABAergic neurons in the lateral globus pallidus (LGP), which project directly towards the pedunculopontine tegmental neurons (PPTg), are activated by prepulse stimuli but not by startle pulse stimuli and play an important role in PPI [38]. It is suggested that the pallidotegmental GABAergic neurons act as an interface between the brainstem PPI-mediating and the forebrain PPI-regulating circuits by using c-Fos, a neural activation marker, immunohistochemistry. Moreover, we demonstrated that the disruption of PPI caused by METH was accompanied by impairment of the LGP and hyperactivation of the caudal pontine reticular nucleus (PnC), which manifested as changes in c-Fos expression in the LGP and PnC after the PPI test; therefore, it is reasonable to assume that METH disrupts PPI of the startle reflex in mice by inhibiting the activation of pallidotegmental GABAergic neurons evoked by a prepulse stimulus [39]. 

Baclofen (1-5 mg/kg) dose-dependently ameliorated PPI impairment induced by acute treatment with METH (3 mg/kg) (Table **1**), which was associated with the reversal of METH-induced decrease in c-Fos expression in LGP, and METH-induced increase in c-Fos expression in PnC. Consistent with our findings, it was reported that baclofen reversed the reduction in PPI induced by MK-801, but not that by apomorphine (a direct dopamine receptor agonist), in rats [40], and that baclofen and clozapine, but not haloperidol, improved spontaneous PPI deficit in mice [41].

## CONCLUSION

In conclusion, baclofen acutely ameliorated the cognitive deficits in repeated METH-treated mice, an animal model for cognitive deficits in METH abuse and schizophrenia. GABA_B_ receptors may constitute a putative new drug target for treating cognitive deficits in these patients. Further studies are necessary to clarify the molecular mechanisms of the action of baclofen.

## Figures and Tables

**Table 1:** Effects of GABA Receptor Agonist on Methamphetamine-Induced Congnitive Dysfunction in Mice

	Methamphetamine-Induced Congnitive Impairment	
	Recongnition Memory	Sensorimotor Gating*
Baclofen	↑	↑
Gaboxadol	±	N.D.

↑: improvement; ± :no effect; N.D.: not detected.

* Sensorimotor gating was assessed by the PPI of the acoustic startle reflex.
